# Comprehensive analysis of tissue proteomics in patients with papillary thyroid microcarcinoma uncovers the underlying mechanism of lymph node metastasis and its significant sex disparities

**DOI:** 10.3389/fonc.2022.887977

**Published:** 2022-08-29

**Authors:** Zhen Cao, Zejian Zhang, Xiaoyue Tang, Rui Liu, Mengwei Wu, Jianqiang Wu, Ziwen Liu

**Affiliations:** ^1^ Department of General Surgery, Peking Union Medical College Hospital, Chinese Academy of Medical Sciences and Peking Union Medical College, Beijing, China; ^2^ Department of Medical Research Center, State Key Laboratory of Complex Severe and Rare Diseases, Peking Union Medical College Hospital, Chinese Academy of Medical Sciences and Peking Union Medical College, Beijing, China

**Keywords:** papillary thyroid microcarcinoma, lymph node metastasis, proteomics, sex, mass spectrometry

## Abstract

**Background:**

Lymph node metastasis (LNM) in papillary thyroid microcarcinoma (PTMC) is associated with an increased risk of recurrence and poor prognosis. Sex has been regarded as a critical risk factor for LNM. The present study aimed to investigate the molecular mechanisms underlying LNM and its significant sex disparities in PTMC development.

**Methods:**

A direct data-independent acquisition (DIA) proteomics approach was used to identify differentially expressed proteins (DEPs) in PTMC tumorous tissues with or without LNM and from male and female patients with LNM. The functional annotation of DEPs was performed using bioinformatics methods. Furthermore, The Cancer Genome Atlas Thyroid Carcinoma (TCGA-THCA) dataset and immunohistochemistry (IHC) were used to validate selected DEPs.

**Results:**

The proteomics profile in PTMC with LNM differed from that of PTMC without LNM. The metastasis-related DEPs were primarily enriched in categories associated with mitochondrial dysfunction and may promote tumor progression by activating oxidative phosphorylation and PI3K/AKT signaling pathways. Comparative analyses of these DEPs revealed downregulated expression of specific proteins with well-established links to tumor metastasis, such as SLC25A15, DIRAS2, PLA2R1, and MTARC1. Additionally, the proteomics profiles of male and female PTMC patients with LNM were dramatically distinguishable. An elevated level of ECM-associated proteins might be related to more LNM in male PTMC than in female PTMC patients. The upregulated expression levels of MMRN2 and NID2 correlated with sex disparities and showed a positive relationship with unfavorable variables, such as LNMs and poor prognosis.

**Conclusions:**

The proteomics profiles of PTMC show significant differences associated with LNM and its sex disparities, which further expands our understanding of the functional networks and signaling pathways related to PTMC with LNM.

## Introduction

Papillary thyroid carcinoma (PTC) is the most common pathological type of malignant thyroid neoplasm and has a favorable prognosis. Among PTCs, tumors measuring ≤ 10 mm in maximal diameter are defined by the World Health Organization (WHO) as papillary thyroid microcarcinoma (PTMC). The development of ultrasonography and fine-needle aspiration biopsy (FNAB) has increased the detection rate of newly diagnosed PTMC. Overall, management of PTMC remains controversial due to its indolent behavior and good prognosis (1, 2). In 2015, active surveillance (AS) was accepted as an alternative management option by the American Thyroid Association (ATA) for patients with low-risk PTMC (3). However, PTMC with high-risk features, such as lymph node metastasis (LNM), extrathyroidal extension (ETE), and invasion to adjacent organs are not suitable for AS and surgery is needed (4). If indicated, postoperative radioactive iodine therapy should be conducted.

In PTMC, the known risk factors associated with LNM include patient age, sex, multifocality, calcification, and ETE (5–7). Sex is an important factor in the pathogenic mechanism, diagnosis, and prognosis of various cancers, particularly sex-specific organs. Sex disparities in cancers of some organs shared by males and females, such as the thyroid, liver, and lung, have also been reported (8). Thyroid cancer is one of the fastest-growing cancers diagnosed worldwide. It is 2.9 times more common in women than in men (9). Sex is a significant parameter for PTC patients, and the clinicopathological features of PTC vary by sex. It has been found that females have an earlier age of onset but that males tend to have higher mortality. Multiple previous studies have suggested that male sex is a risk factor for LNM in PTC or PTMC (10–12). Nevertheless, sex disparities are still rarely explored in thyroid cancer, and sex differences remain largely underestimated when considering clinical therapeutic strategies.

Liquid chromatography-tandem mass spectrometry (LC-MS/MS) is an effective and empirical method for simultaneous identification and quantification of proteins in different samples. As the proteome determines the function of a cell, the application of proteomics is a feasible and significant strategy for comprehensively analyzing differences in protein expression and elucidating the process underlying diseases manifestations. The usage of LC-MS/MS approaches is regarded as a breakthrough in proteomics. Multiple published works in thyroid cancer proteomics have targeted PTC and focused on comparing the protein profiles of PTC tissues with those of benign or healthy tissues (13, 14). Nevertheless, the protein profiles of LNM and its sex disparities in PTMC patients are still unknown, and such information might provide a reasonable basis for investigating sex as a risk factor for LNM.

Therefore, to unveil differentially expressed proteins (DEPs) that indicate sex disparities of LNM in PTMC, we applied the direct data-independent acquisition (DIA) proteomics technique to analyze the protein profiles of neoplastic and peritumoral tissues. By comparing LNM versus non-LNM (NLNM) and male PTMC with LNM (MLNM) versus female PTMC with LNM (FLNM) thyroid cancer tissues, the crucial DEPs were identified to reveal the underlying mechanisms of the differences at the protein level using bioinformatics analysis. This study provides novel insight into sex disparities of LNM in PTMC.

## Materials and methods

### Sample collection and study design

Neoplastic tissues and peritumoral tissues were collected from 72 PTMC patients recruited at the Peking Union Medical College Hospital (PUMCH, Beijing, China) from September 2020 to January 2021. The inclusion criteria were as follows: (1) maximal diameter of the thyroid nodule ≤ 10 mm; (2) age ≥ 18 years at the time of surgery; and (3) diagnosed based on thyroid ultrasonography, FNA and postoperative pathology, as confirmed by two pathologists. The exclusion criteria were as follows: (1) autoimmune, blood, or infectious diseases; (2) a history of previous thyroid surgery or other malignancy surgery or a history of chemotherapy or radiotherapy. Meanwhile, because diabetes may increase the risk of thyroid cancer as a potential confounding factor, patients with diabetes or impaired glucose tolerance were also excluded from this study. This study was approved by the Ethics Committee of PUMCH (No. JS-2670).

**Figure 1:**
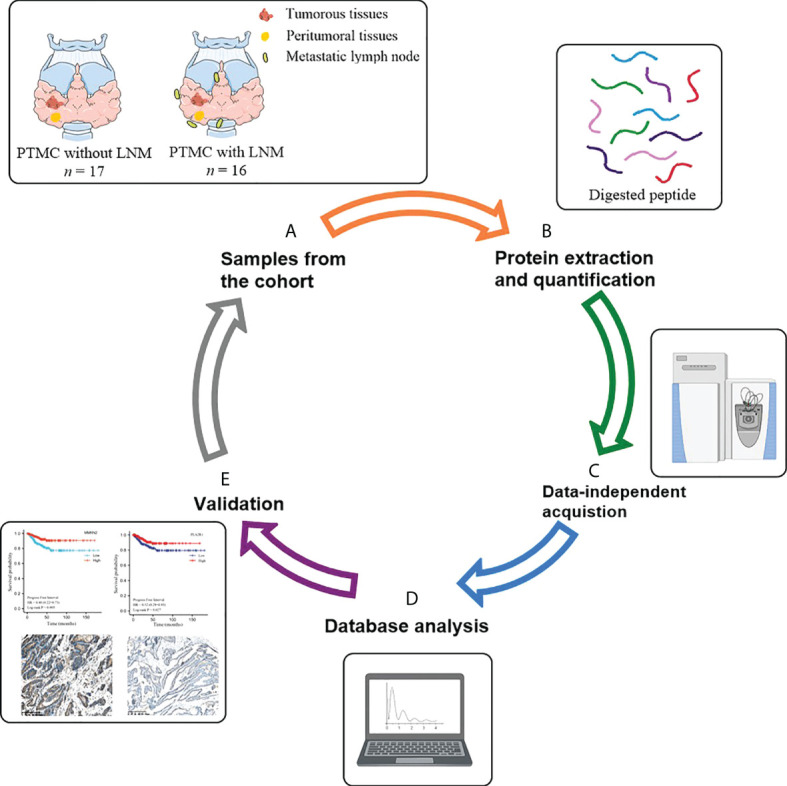
Schematic diagram of the experimental workflow employed in this study. **(A)** Collection of neoplastic tissues and peritumoral tissues from PTMC patients; **(B)** High-throughput sample including tissue grinding and lysis, and protein extraction and quantification; **(C)** Direct data-independent acquisition analysis of tissue proteomics and data processing; **(D)** Bioinformatics and statistical analyses; **(E)** Validation. PTMC, papillary thyroid microcarcinoma; LNM, lymph node metastasis.

The flow chart of the study design is depicted in **Figure 1**. In the discovery phase, tumor tissues from 17 PTMC patients without LNM were included in the NLNM group, and tumor tissues from 16 PTMC patients with LNM were included in the LNM group. Peritumoral tissues from these 33 patients were randomly mixed into 6 pooled samples as the control group. Tissue proteomes were compared among patients with LNM versus NLNM and MLNM versus FLNM to obtain a comprehensive overview of DEPs associated with LNM and sex differences. The clinical information of these patients is listed in **Table 1**. In the validation phase, IHC was performed on the tumorous and peritumoral tissues from another 23 PTMC patients (12 NLNM and 11 LNM patients) to verify the proteomic results for LNM. IHC was also performed on the tumorous tissues of another 16 PTMC patients with LNM (8 males and 8 females) to verify the proteomic results for sex disparities in LNM. The detailed clinical information of these 39 patients is listed in **Supplementary Table 1**.

**Table 1:** Clinical and pathological characteristics of the subjects used for proteomics analysis.

	PTMC without LNM (n = 17)	PTMC with LNM (n = 16)	*P*-value
Age at operation [y, $\bar{\text{x}}$ ± S]	40.59 ± 8.09	37.94 ± 7.56	0.339
BMI [kg/m^2^, $\bar{\text{x}}$ ± S]	24.12 ± 3.01	23.50 ± 2.14	0.503
Sex [n (%)]			0.119
Female	12 (70.6)	7 (43.8)	
Male	5 (29.4)	9 (56.2)	
Family history of thyroid disease [n (%)]			0.758
No	12 (70.6)	13 (81.3)	
Yes	5 (29.4)	3 (18.7)	
Tumor size [cm, $\bar{\text{x}}$ ± S]			
≤ 0.5	8 (47.1)	4 (25.0)	0.188
> 0.5	9 (52.9)	12 (75.0)	
Clinical LNM [n (%)]			0.227
Absent	17 (100.0)	14 (87.5)	
Present	0 (0.0)	2 (12.5)	
Pathological subtype [n (%)]			0.356
Classic	11 (64.7)	13 (81.3)	
Follicular variant	4 (23.5)	1 (6.2)	
Classic and follicular variant	2 (11.8)	2 (12.5)	
Tumor location [n (%)]			0.392
Unifocal	12 (70.6)	9 (56.2)	
Multifocal	5 (29.4)	7 (43.8)	
Tumor calcification [n (%)]			0.712
Absent	4 (23.5)	2 (12.5)	
Present	13 (76.5)	14 (87.5)	
Microscopic capsular invasion [n (%)]			0.226
Absent	5 (29.4)	8 (50.0)	
Present	12 (70.6)	8 (50.0)	
Side of lobectomy [n (%)]			0.881
Unilateral	7 (41.2)	7 (43.8)	
Bilateral	10 (58.8)	9 (56.2)	
Hashimoto’s thyroiditis [n (%)]			0.758
Absent	12 (70.6)	13 (81.3)	
Present	5 (29.4)	3 (18.7)	

PTMC, papillary thyroid microcarcinoma; LNM, lymph node metastasis; BMI, body mass index. Data are presented as mean ± standard deviation, or n (%).

### Protein extraction and quantification

Neoplastic and peritumoral tissues (80-120 mg) were cut and washed three times with cold phosphate-buffered saline (PBS) to eliminate blood contamination. The tissues were homogenized with cold lysis buffer (8M urea in PBS, pH 8.0, 1 mM phenylmethanesulfonyl fluoride [PMSF], 1 × cocktail) using a Q800R3 sonicator (Qsonica, Newtown, CT, USA). After centrifugation at 12,000 rpm for 15 min at 4°C, the supernatant was transferred to a tube, and the protein concentration was determined using a Nanodrop 2000 (Thermo Scientific, Branchburg, NJ, USA). Each tissue protein sample (100 µg/µL) was treated with 10 mM dithiothreitol (DTT) at 55°C for 30 min and alkylated with 25 mM iodoacetamide (IAA) at room temperature in the dark for 30 min. The proteins were digested with trypsin/Lys-C mixture at a protein/protease ratio of 50:1 at 37°C overnight. The digested protein sample was desalted, dried, and dissolved in 20 µL of 0.1% formic acid (FA) for subsequent LC-MS/MS analysis.

### DIA proteomics analysis

LC-MS/MS analysis was performed using an EASY-nLC 1200 UHPLC system and Q-Exactive HF (Thermo Scientific, Rockwell, IL, USA). All peptides dissolved in 0.1% FA were loaded onto the trap column. Subsequently, the eluent was transferred to a reversed-phase analytical column (75 μm × 500 mm, 2 μm, MONOTECH). The elution gradient was 5-30% buffer B (flow rate = 300 nl/min; 0.1% FA in 99.9% acetonitrile) over 120 min. An iRT kit (Biognosys AG, Schlieren, Switzerland) was used for retention time alignments in all samples. The parameters of MS were set as follows: the full scan was achieved by a resolution of 120,000 and in the range of 400-1,200 m/z; the cycle time was set at 3 s; the AGC was 3e6; the injection time was under 100 ms; charge state screening was performed by precursors with a +2 to +6 charge state; and the dynamic exclusion duration was 10 s. According to the precursor m/z distribution of the pooled sample, the number of precursor ions in each isolation window was equalized.

### Proteomics data analysis

The raw data from DIA proteomics analysis were searched using Spectronaut Pulsar X (Biognosys, AG, Schlieren, Switzerland) software with default settings. The mass spectrometry proteomics data have been deposited at ProteomeXchange Consortium (http://proteomecentral.proteomexchange.org) *via* the iProX partner repository with the dataset identifier PXD031838. An optimal XIC extraction window was identified based on the iRT calibration strategy. The mass tolerance strategy was set to dynamic based on extensive mass calibration. The cross-run normalization was set to local normalization based on local regression. The sum peak areas of the respective fragment ions in MS_2_ were used to quantify peptide intensities. The k-nearest neighbors (KNN) method was used to fill in missing values of protein abundance.

### Bioinformatics analyses

For proteomics analysis of neoplastic and peritumoral tissues, a 1.5-fold change was set as the threshold for DEPs. Principal component analysis (PCA) and orthogonal partial least squares discriminant analysis (OPLS-DA) were conducted using SIMCA software (version 14.1, Umetrics, Sweden). Gene Ontology (GO) functional enrichment analysis was performed using the R program (Version 3.5.1, R Foundation for Statistical Computing, Vienna, Austria) with the “clusterProfiler” package. A false discovery rate (FDR) < 0.05 was set as the threshold for statistical significance in GO enrichment analysis. For Ingenuity Pathway Analysis (IPA, version 2.3; Qiagen, CA, United States), all DEPs were imported to analyze pathways, diseases, and functions. Protein-protein interaction (PPI) network analysis was conducted using the Search Tool for the Retrieval of Interacting Genes (STRING) database (http://string-db.org) (15) and visualized in Cytoscape (version 3.7.1, Cytoscape Consortium, New York, NY, USA).

We used the Genomic Data Commons (GDC) API to download level-3 RNA sequencing (RNA-seq) data from The Cancer Genome Atlas Thyroid Carcinoma (TCGA-THCA) database, including 507 PTC cases and relevant follow-up information. Transcript per million (TPM) transformation followed by base-2 logarithm normalization was applied. Given the shallow cancer-related death rate, progression-free interval (PFI) data from the University of California Santa Cruz (UCSC) Xena database were extracted as the specific survival outcome (16). Both structural evidence and biochemical evidence of recurrence were defined as progression. After removing duplicate samples, differential gene expression analysis of 452 PTC cases from the TCGA-THCA dataset was performed by using the R package “DESeq2” (17). According to the criteria of FDR < 0.05 and |log2 fold change (log2FC) | > 0.58, differentially expressed genes (DEGs) were identified. Kaplan-Meier plots were generated for PFI using the “survminer” and “survival” R packages.

### Statistical analysis

All statistical analyses and visualizations were conducted with SPSS statistics (version 25.0, IBM Corp., Armonk, NY, USA) and GraphPad Prism (version 8.0.2, GraphPad Software Inc, San Diego, USA). Comparisons of survival curves were analyzed with the log-rank (Mantel-Cox) test. Categorical variables were analyzed by the chi-square test. Continuous variables were expressed as mean and SD and compared using the independent sample t test (normal distribution) or the Mann-Whitney U test (non-normal distribution). Two-sided *P* < 0.05 was considered statistically significant.

### Immunohistochemistry (IHC) validation

Tissue sections (5 μm) were dewaxed at 60°C for 30 min, followed by two 5-min washes with xylene. Then, the sections were rehydrated by sequential 5-min washes in 100%, 95%, and 80% ethanol and distilled water. Antigen retrieval was performed by heating the tissues at 95°C for 10 min in sodium citrate buffer (0.01 M, pH 6.0). The endogenous peroxidase activity of the tissue was blocked by 3% hydrogen peroxide for 30 min, followed by incubation with primary detection antibodies overnight at 4°C. The sections were incubated with the Polink-2 Plus^®^ HRP Polymer Detection System (PV-9001 and PV-9002; ZSGB-BIO) according to the manufacturer’s instructions. The samples were developed using 3, 3′-diaminobenzidine (DAB) substrate (Dako) and counterstained with hematoxylin.

IHC staining was evaluated based on the percentage and intensity. The percentage of positive cells was scored as 1–4 (1 = 1%~25%, 2 = 26%~50% cells, 3 = 51%~75% cells, and 4 = 76%~100% cells). The staining intensity of positive cells was scored as 0–3 (0 = no staining, 1 = weak staining, 2 = moderate staining, and 3 = strong staining). The final scores (ranging from 0 to 12) were obtained by multiplying the percentage and intensity scores.

## Results

### Flow chart of the study

Tissue proteomes were compared among patients with LNM versus NLNM and MLNM versus FLNM to obtain a comprehensive description of the DEPs associated with LNM and sex. The flow chart of the study design is shown in **Figure 1**. The protein profiles of tumorous tissues and peritumoral tissues were determined using the direct DIA proteomics technique. Overall, 5,005 credible proteins with at least two unique peptides were obtained in 45 samples (**Supplementary Table 2**). The key DEPs were screened, and bioinformatics analyses were performed.

### Functional and biological pathway analyses of DEPs in PTMC tumorous tissues with LNM compared to those with NLNM

**Figure 2:**
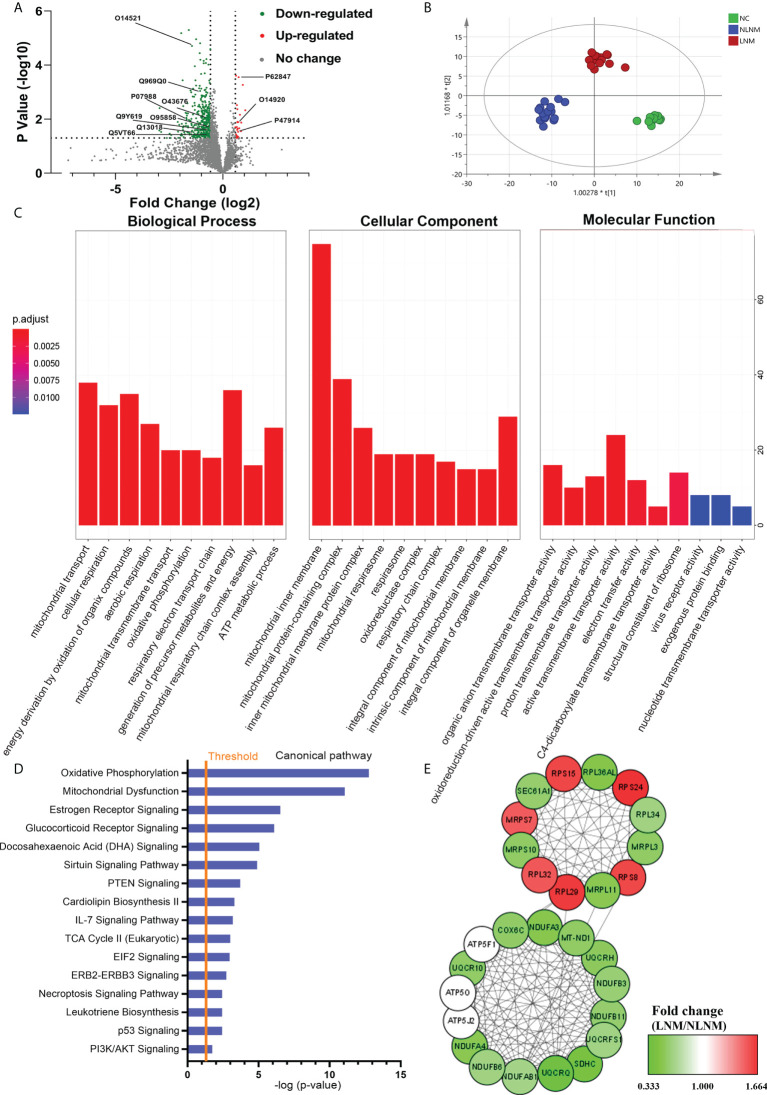
Comparative analysis of proteome expression profiles between the LNM and NLNM groups. **(A)** Scatter plot showing the distribution of downregulated (green dots) and upregulated (red dots) differentially expressed proteins (DEPs) between the LNM and NLNM groups. **(B)** Orthogonal partial least squares discriminant analysis revealed differences in the proteome profiles among the LNM, NLNM, and normal control (NC) groups. “LNM” refers to PTMC with lymph node metastasis, “NLNM” refers to PTMC without lymph node metastasis, and “NC” refers to peritumoral tissues. **(C)** Gene Ontology enrichment analysis of DEPs between the LNM and NLNM groups. The categories of biological process (BP), cellular component (CC) and molecular function (MF) are respectively shown. **(D)** Annotation and functional characterization of DEPs. **(E)** Protein-protein interaction (PPI) network analysis of DEPs between the LNM and NLNM groups.

To comprehensively discover the molecular mechanisms involved in the pathogenesis of PTMC susceptibility to LNM, global protein profiles from PTMC patients with LNM and NLNM were obtained. Using a 1.5-fold change cut-off for the classification of differential expression, a total of 411 DEPs in LNM versus NLNM were identified (*P* < 0.05), with 26 upregulated and 385 downregulated proteins (**Figure 2A**; **Supplementary Table 3**). OPLS-DA was performed, and the score plots showed that LNM, NLNM, and NC could be separated from each other in the model (**Figure 2B**). Further GO analysis indicated that these DEPs mainly participated in the biological processes of mitochondrial transport, cellular respiration, and energy derivation *via* the oxidation of organic compounds (**Supplementary Table 4**). In terms of cellular components, the DEPs showed significant enrichment in the mitochondrial inner membrane, mitochondrial protein-containing complex, and inner mitochondrial membrane protein complex. In addition, these DEPs were primarily related to the molecular function of active transmembrane transporter activity (**Figure 2C**). The GO enrichment analysis was performed separately on upregulated and downregulated proteins (**Supplementary Figures 1A–F**).

To gain insight into the potential role that these DEPs may play in the functional characterization of LNM progression, IPA was performed on all 411 DEPs. Canonical pathway analysis indicated predominantly enriched of multiple metastasis-related pathways, such as the oxidative phosphorylation pathway, mitochondrial dysfunction pathway, IL-7 signaling pathway, and PI3K/AKT signaling pathway (**Figure 2D**). The pathway enrichment analysis was also performed separately on downregulated and upregulated proteins (**Supplementary Figures 1G, H**). To further place the identified proteins within the context of known PPI and gain insights into the coordinated roles of these proteins, the interactions between all DEPs were investigated using the online resource STRING, and PPI networks were visualized in Cytoscape based on their STRING score. We identified two main clusters, as illustrated in **Figure 2E**, which correspond to two main functions: oxidative phosphorylation and structural constituent of ribosome.

### External validation of DEPs between LNM and NLNM using TCGA-THCA and IHC

**Figure 3:**
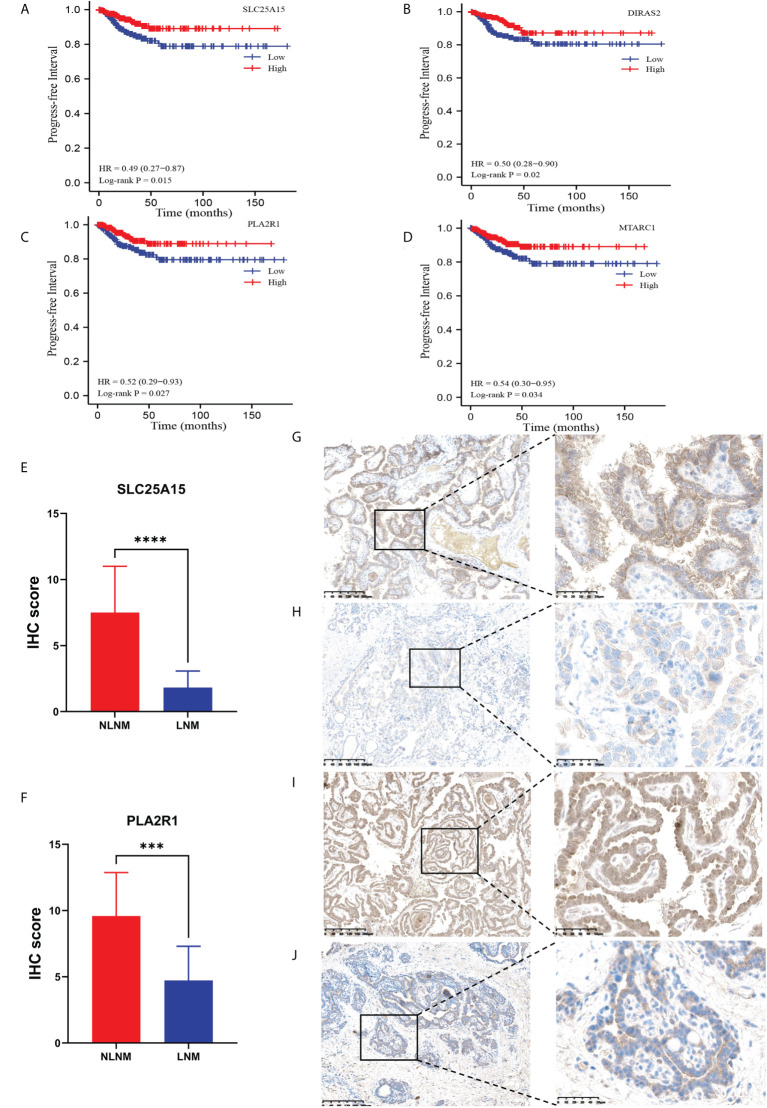
Kaplan–Meier survival curves (log-rank test) showing the correlation between progression-free survival and DEP expression (SLC25A15, DIRAS2, PLA2R1, and MTARC1). **(A)** SLC25A15 mRNA expression in thyroid tumorous tissues is positively related to poor prognosis (log-rank test *P* = 0.015); **(B)** DIRAS2 mRNA expression in thyroid tumorous tissues is positively associated with poor prognosis (log-rank test *P* = 0.020); **(C)** PLA2R1 mRNA expression in thyroid tumorous tissues is positively associated with poor prognosis (log-rank test *P* = 0.027); **(D)** MTARC1 mRNA expression in thyroid tumorous tissues is positively associated with poor prognosis (log-rank test *P* = 0.034); **(E, F)** Validation of the differential expression of SLC25A15 and PLA2R1 between LNM and NLNM in another independent cohort. IHC staining was assessed as the sum of the percentage and intensity scores. (***) *P*<0.001, (****) *P*<0.0001. **(G–J)** Representative IHC staining of SLC25A15 **(G**, NLNM; **H**, LNM**)** and PLA2R1 **(I**, NLNM; **J**, LNM**)** in tumorous tissues.

To confirm the accuracy of our data as a resource for further study, it is necessary to conduct secondary validation. In total, we identified 411 DEPs in LNM versus NLNM. Through DEG analysis of 452 PTC cases from the TCGA-THCA dataset, including 229 PTC patients with NLNM and 223 PTC patients with LNM, we retrieved a total of 3,446 DEGs. The intersection contained 23 DEGs (**Table 2**). Kaplan-Meier survival analyses were used to assess the prognostic value of the 23 DEGs between NLNM and LNM in the TCGA-THCA dataset. The results indicated a significant correlation with PFI ability for SLC25A15, DIRAS2, PLA2R1, and MTARC1 (log-rank *P* = 0.015, *P* = 0.020, *P* = 0.027 and *P* = 0.034) (**Figures 3A–D**). We also assessed the status of these four DEPs using RNA-seq data from the TCGA-THCA dataset. Comparisons between LNM and NLNM tissues showed mRNA expression of SLC25A15, DIRAS2, PLA2R1, and MTARC1 to be downregulated in LNM tissues, which was consistent with our proteomics data. Then, two DEPs (SLC25A15 and PLA2R1) were further validated in another independent cohort of 23 PTMC patients using the IHC method. The results confirmed these two proteins to be significantly downregulated in LNM tissues compared to NLNM tissues (**Figures 3E, F**). Typical IHC staining images are shown in **Figures 3G–J**. Two DEPs (SLC25A15 and PLA2R1) were also validated their expression difference between tumor and peritumoral tissues using IHC method. The IHC results is shown in **Supplementary Figures 2A, B**. Typical IHC staining images are shown in **Supplementary Figures 2C–F**.

**Table 2:** Intersection containing 23 DEGs associated with LNM.

Protein Groups	Gene name	Metastasis DEPs in our data		Metastasis DEGs in TCGA	
		Log2FC	*P*-value	Log2FC	*P*-value
O15020	SPTBN2	-0.88105	0.021632	0.586635	6.2E-07
O75309	CDH16	-1.03375	0.024093	-1.92791	3.17E-19
O76076	CCN5	-0.8827	0.015232	0.910836	3.03E-06
P00491	PNP	0.659164	0.041600	0.820436	6.9E-10
P07988	SFTPB	-1.69991	0.008995	1.240076	1.01E-08
P08174	CD55	-1.04209	0.013791	0.719739	6.51E-08
P10415	BCL2	-1.62324	0.006101	-0.65017	4.6E-15
P10515	MUC1	-0.87371	0.004778	0.759667	1.22E-06
P10586	ITGA2	-1.81713	0.018013	0.624354	4.21E-07
P24310	COX7A1	-1.3813	0.019440	-0.69593	1.02E-07
P24539	CHI3L1	-0.65926	0.024904	1.246438	8.3E-10
P51674	GPM6A	-1.0253	0.033932	-0.83176	3.02E-09
P51688	PRRX1	-0.61801	0.023193	0.615458	3.57E-07
Q13018	PLA2R1	-1.3703	0.030249	-0.80338	1.24E-10
Q13084	LAMB3	-0.96885	0.020498	0.731767	1.32E-06
Q13423	DSC3	-0.68046	0.009219	0.806844	0.00034
Q30154	HLA-DRB5	-1.4606	0.014741	0.621312	1.07E-05
Q32NB8	MTARC1	-0.81087	0.002417	-0.633	2E-14
Q8TF66	LRRC15	-0.77483	0.028112	1.693971	8.42E-12
Q8WTS1	SPOCK2	-1.85866	0.025249	0.640279	1.69E-05
Q96HU8	DIRAS2	-0.62135	0.048988	-1.23397	1.38E-18
Q9ULD0	OGDHL	-0.74369	0.038228	-1.35033	6.4E-22
Q9Y619	SLC25A15	-0.68524	0.024124	-1.07027	6.16E-21

### Functional and pathway analyses of DEPs in PTMC tumorous tissues with LNM according to sex

**Figure 4:**
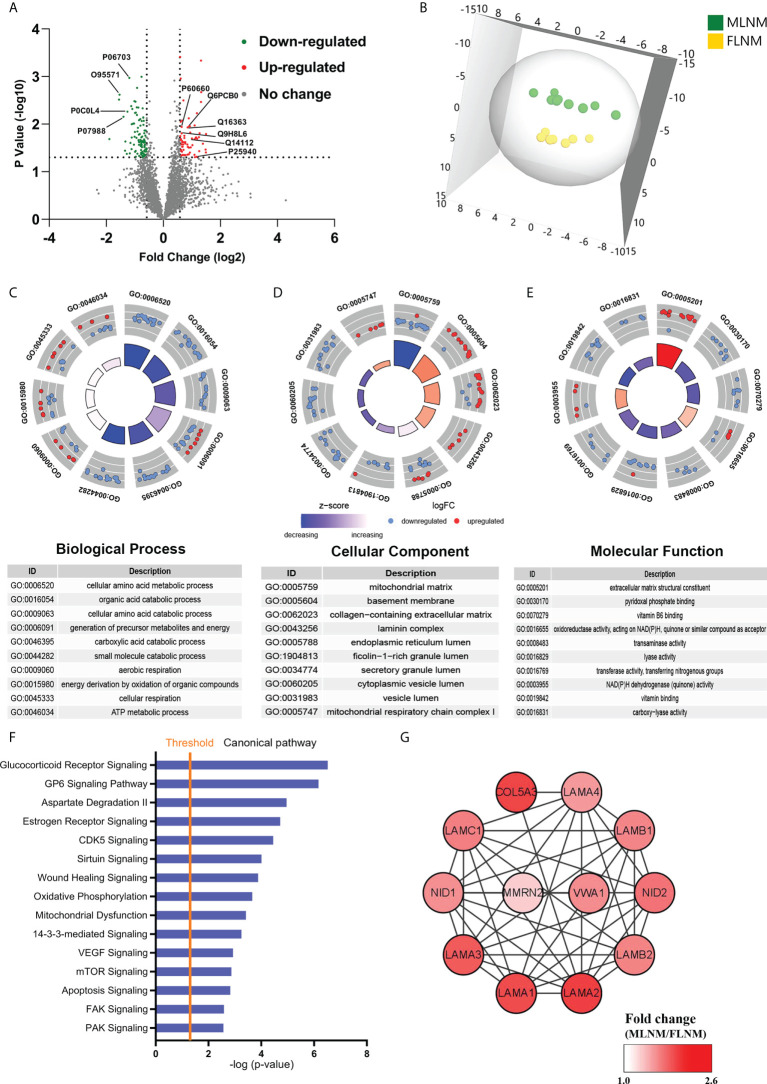
Comparative analysis of the proteome expression profiles between the MLNM and FLNM groups. **(A)** Scatter plot showing the distribution of downregulated (green dots) and upregulated (red dots) differentially expressed proteins (DEPs) between the MLNM and FLNM groups. **(B)** Orthogonal partial least squares discriminant analysis revealed differences in the proteome profiles between the MLNM and FLNM groups. “MLNM” refers to male PTMC patients with lymph node metastasis, and “FLNM” refers to female PTMC patients with lymph node metastasis. **(C–E)** Gene Ontology analysis revealed enrichment of these DEPs in the biological process (BP), cellular component (CC) and molecular function (MF) categories; **(F)** Annotation and functional characterization of DEPs. **(G)** The ECM-related DEPs in the protein-protein interaction (PPI) network are shown as nodes. The intensity of the red colors indicates the ratio of protein abundance in MLNM/FLNM.

To comprehensively explore the significance of the molecular mechanisms of sex disparities in PTMC with LNM, the differences in protein expression between MLNM and FLNM were compared. A total of 169 DEPs (fold change > 1.5; *P* < 0.05) were identified consisting of 64 upregulated and 105 downregulated proteins (**Figure 4A**; **Supplementary Table 5**). The proteomic profiling OPLS-DA was performed based on these DEPs, and the score plot showed a tendency to separate MLNM tissue from FLNM tissue (**Figure 4B**).

GO enrichment analyses were also performed on these 169 DEPs to further explore the molecular mechanisms involved in sex disparities of LNM (**Supplementary Table 6**). Many of the DEPs showed enrichment in the cellular amino acid metabolic process, organic acid catabolic process, and cellular amino acid catabolic process in the biological process category (**Figure 4C**). In the cellular component category, the top enriched categories were related to the mitochondrial matrix, basement membrane, and collagen-containing extracellular matrix (ECM) (**Figure 4D**). Molecular function analysis revealed that these proteins were mainly located in ECM structural constituents (**Figure 4E**). The GO enrichment analysis was performed separately on upregulated and downregulated proteins (**Supplementary Figures 3A–F**). To gain a better understanding of the biological pathways in MLNM, IPA was conducted on the 169 DEPs. Numerous signaling pathways associated with the DEPs were identified, including glucocorticoid receptor (GR) signaling pathway, GP6 signaling pathway, estrogen receptor signaling pathway, CDK5 signaling pathway, VEGF signaling pathway, and mTOR signaling pathway (**Figure 4F**). The pathway enrichment analysis was also performed separately on downregulated and upregulated proteins (**Supplementary Figures 3G, H**).

To gain a better overview of the DEPs between MLNM and FLNM, comprehensive PPI networks were constructed using Cytoscape 3.7.1 to visualize potential relationships among these proteins. An intricate network of PPIs that indicated the closest core interactions among these DEPs is shown in **Figure 4G**. According to the map, LAMA1, LAMA2, LAMA3, LAMA4, LAMB1, LAMB2, LAMC1, NID1, NID2, COL5A3, MMRN2, and VWA1 were upregulated.

### External validation of DEPs between MLNM and FLNM using TCGA-THCA and IHC

**Figure 5:**
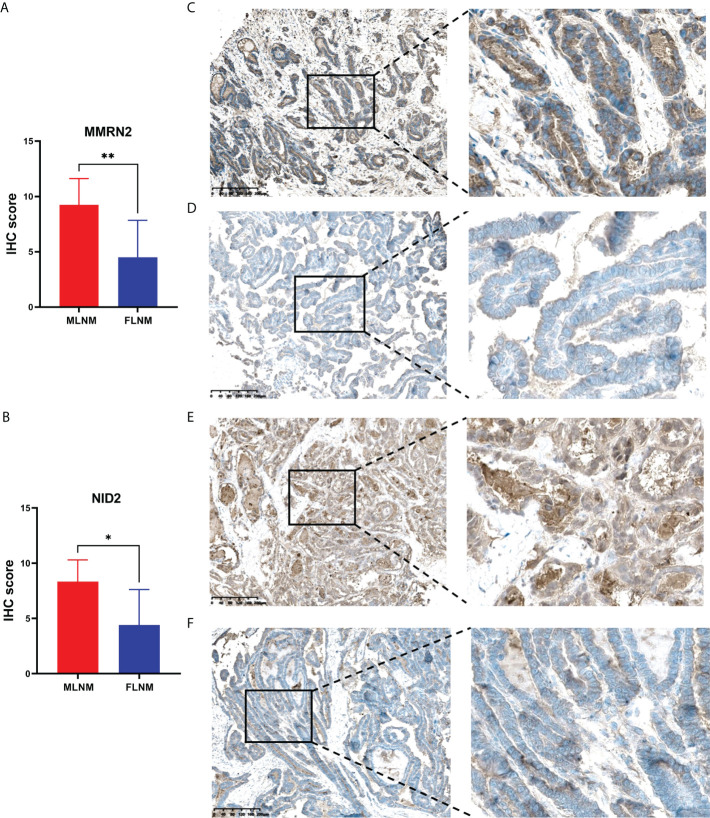
Validation of the differential expression of MMRN2 and NID2 between MLNM and FLNM in another independent cohort. **(A, B)** MMRN2 and NID2 were significantly upregulated in MLNM tissues compared with FLNM tissues. IHC staining was assessed as the sum of the percentage and intensity scores. (*) *P*<0.05, (**) *P*<0.01. **(C–F)** Representative IHC staining of MMRN2 (**C**, MLNM; **D**, FLNM) and NID2 (**E**, MLNM; **F**, FLNM) in PTMC tissues.

LNM and tumor recurrence after initial surgery are the main factors associated with poor outcomes for PTMC patients. Moreover, male sex is a known risk factor for LNM in PTMC and males tend to have higher mortality. Hence, we further screened prognosis-related proteins from DEPs related to sex disparities. Considering the relatively good prognosis of PTMC and extremely low risks associated with overall survival, the progression-free interval (PFI) was chosen as the primary endpoint. TCGA-THCA dataset was used to determine whether the DEPs were associated with PFI. Kaplan-Meier survival analyses were applied to evaluate the prognostic value of the 12 upregulated DEPs between MLNM and FLNM in the TCGA-THCA dataset. We found that MMRN2 and NID2 showed significant correlations with PFI (log-rank *P* = 0.003 and *P* = 0.037, respectively). Then, we further validated the expression of MMRN2 and NID2 in another independent cohort of 16 PTMC patients using the IHC method. The IHC staining results confirmed MMRN2 and NID2 to be significantly upregulated in MLNM tissues compared to FLNM tissues (**Figures 5A, B**). Typical images of staining are shown in **Figures 5C–F**.

## Discussion

Although differences in PTC versus benign and PTC versus healthy thyroid tissue have been widely described, differences in protein levels between male PTMC patients with LNM and female PTMC patients with LNM have not yet been analyzed using proteomics techniques (18, 19). In our study, the molecular mechanisms underlying metastasis-related differences and sex differences in LNM were investigated. A comprehensive description of the changes in the protein profiles of PTMC with LNM and its sex disparities was produced. Using direct-DIA proteomics analysis, we obtained 5,005 credible proteins from 45 specimens and observed clear separation of the protein profiles in PTMC tumorous tissues with peritumoral tissues. Furthermore, the protein profiles of peritumoral tissues were prominently similar, which agreed with the findings of previous studies (20). These results reveal that the tumor microenvironment has little effect on peritumoral tissues. Hence, our further studies focused mainly on DEPs in PTMC tumorous tissues.

Consistent with the different pathologic features found, there was a distinct difference in DEPs between LNM and NLNM. We next performed GO and IPA analyses of DEPs. Interestingly, the DEPs were found to be mainly enriched in the mitochondrial inner membrane, mitochondrial protein-containing complex, mitochondrial transport, and cellular respiration. The most connected DEPs were closely related to mitochondrial dysfunction. These results indicated that mitochondrial dysfunction-associated proteins might play a more significant role in LNM than in NLNM. As vital organelles, mitochondria play central roles in tumorigenesis, cellular Ca^2+^ homeostasis, and apoptosis, which affect cellular signaling pathways. Active mitochondrial remodeling and adaptation of cancer cells could control the tumor microenvironment and influence the molecular characteristics of tumor cells heterogeneitys (21). Metastatic cancer cells undergo enhanced mitochondrial respiration for energy generation, which produces excess reactive oxygen species (ROS) and oxidizes mitochondrial DNA and proteins, altering mitochondrial stability and triggering tumorigenesis (22). Dysregulation of mitochondrial functions in epithelial tumor cells and tumor-associated stroma may promote metastasis formation (23). Combined with the results of our study, it can be inferred that mitochondrial dysfunction-associated proteins are mainly related to LNM.

In this study, IPA analysis of the DEPs between LNM- and NLNM- enriched pathways showed them to be associated with multiple metastasis-related pathways, including oxidative phosphorylation (OXPHOS), mitochondrial dysfunction, IL-7 signaling and PI3K/AKT signaling pathway. Increased ATP through the OXPHOS pathway might promote cell detachment and migration energy. Increased ROS through this pathway may promote cell motility. Previous studies have indicated that mitochondrial dysfunction could enhance mitochondrial ROS generation and redox rebalance to stimulate tumor cell proliferation and invasiveness (24). Thus, mitochondrial dysfunction plays an important role in tumor progression. A growing body of evidence indicated that IL-7 could promote the growth and metastasis of malignancies. Jian *et al.* found that IL-7 could upregulate the expression of PI3K and promote the phosphorylation of AKT to activate the PI3K/AKT signaling pathway (25). AKT has been reported to rapidly accumulate in mitochondria following PI3K activation and regulate cell proliferation, apoptosis, angiogenesis, and the cell cycle by activating downstream cell receptors (26, 27). Furthermore, activation of the PI3K/AKT pathway by the IL-7 signaling pathway has been confirmed to play a pivotal role in cancer cell proliferation. All these results indicate that OXPHOS, IL-7 signaling, and PI3K/AKT signaling pathways are strongly related to PTMC with LNM. Furthermore, the two main clusters in the PPI network showed close interactions between these identified proteins, which correspond to two broad functions, namely, OXPHOS and structural constituent of ribosome. As mentioned above, OXPHOS could provide energy needed for cell detachment and migration and thereby promote metastasis. We also found that most of the upregulated proteins were from the same family, ribosomal proteins (RPs). RPs are the main components of ribosomes and are involved in the self-assembly of ribosomes and protein synthesis (28). According to published literature, the increased RP content in epithelial cells could contribute to their enhanced metastatic potential (29). Likewise, Lee et al. demonstrated that the high level of mitochondrial RP L44 expression was associated with the presence of lymph node metastasis in PTC, suggesting that it may be a useful clinical marker for predicting the poor prognosis (30). Similarly, another study indicated that ribosome biogenesis regulator homolog (RRS1) silencing could inhibit cell proliferation and promote apoptosis in PTC (31). RRS1 was found as a regulatory protein for ribosome biogenesis. Overall, the RP family might play a vital role in promoting tumorigenesis and metastasis.

To confirm the accuracy of our data as a resource for further study, the metastasis-related DEPs identified in our data were validated with regard to metastasis-related DEGs in TCGA-THCA cohort. Furthermore, four proteins (SLC25A15, DIRAS2, PLA2R1, and MTARC1) related to poor prognosis assessed by the PFI data were downregulated in LNM tissues. IHC analyses were performed for further validation in another cohort of PTMC patients, confirming the significant downregulation of SLC25A15 and PLA2R1 in LNM compared to NLNM tissues. Solute carrier family 25 member 15 (SLC25A15), a mitochondrial ornithine transporter, is critical for normal mitochondrial function. The downregulation of SLC25A15 can facilitate pyrimidine synthesis *via* dihydroorotase and enhances cell proliferation and tumor growth (32, 33). DIRAS family GTPase 2 (DIRAS2), encoding RAS-related small G-proteins, belongs to a branch of the RAS superfamily. Downregulation of DIRAS2 plays a pivotal role in cancer progression and predicts poor prognosis in tumors (34). It has been reported that DIRAS2 was downregulated in ovarian cancer and was associated with decreased overall and disease-free survival, which was consistent with the findings of our study (35). Phospholipase A2 Receptor 1 (PLA2R1) is a transmembrane protein of the mannose receptor family that can regulate several tumor-suppressive responses *via* JAK2 activation (36). Functional experiments have demonstrated that PLA2R1 controls cancer cell death by influencing mitochondrial biology. Previous studies have also demonstrated that the downregulation of PLA2R1 is related to thyroid, breast, and kidney cancers (37, 38). Mitochondrial amidoxime reducing component 1 (MTARC1) is a nitric oxide synthase attached to the mitochondrial membrane that is associated with increased antioxidant capacity, which can be considered the response to counteract augmented oxidative stress (39). MTARC2, which shares a high degree of sequence similarity with MTARC1, was downregulated in human hepatocellular cancer tissues and cells (40, 41). Collectively, these prior studies support the reliability of our proteomics data. Interestingly, SLC25A15, DIRAS2, PLA2R1, and MTARC1 are associated with mitochondrial dysfunction to some extent and have potential as candidate biomarkers for predicting PTMC metastasis and clinical prognosis. Parallel reaction monitoring (PRM) is a targeted proteomic technique. PRM can provide quantitative analysis of the targeted peptides from the rest of FNAB samples and obtain the expression level of these four proteins. This method can be used to detect these candidate biomarkers for preoperatively predicting clinical prognosis.

Compared to female PTMC patients with LNM, the DEPs in the tumorous tissues of male PTMC patients with LNM were mostly enriched in the categories of ECM structural constituent, basement membrane (BM) and collagen-containing ECM. The most connected DEPs were related to the ECM, indicating that ECM-related proteins might be more critical in MLNM than in FLNM. Multiple previous studies reported that dysregulation of the ECM promoted the formation of invasive lesions and led to tumor cell invasion by driving focal adhesions formations (42, 43). Combined with our results, we conclude that an elevated level of ECM-associated proteins might be related to more LNM in male PTMC than in female PTMC patients. The PPI network revealed the close interactions among these identified proteins, and 12 upregulated proteins were associated with ECM function. Interestingly, many upregulated proteins belong to the laminin family. Laminins, the major biologically active component, are significantly involved in the survival and proliferation of cancer cells, malignant phenotype, angiogenesis, and development of premetastatic niches at many stages of cancer progression. Previous studies have shown the prognostic value of laminins in a range of cancers, including gastric (44), lung (45), and endometrial (46) cancer. Moreover, Galatenko *et al.* reported that laminin expression profile analysis was more helpful for colorectal cancer prognosis than single-gene expression analysis (47). Thus, the laminin family proteins identified in our study might play an important role in facilitating metastasis and serve as a biomarker panel for PTMC prognosis.

IPA analysis of the DEPs between MLNM and FLNM showed enrichment in the GR signaling, GP6 signaling, CDK5 signaling, VEGF signaling, and mTOR signaling pathway. GR belongs to the steroid/thyroid/retinoic acid nuclear receptor superfamily. A previous study showed that GR signaling activated TEA domain transcription factors to promote tumor initiation and progression in breast cancer (48). Mechanistically, GR signaling affects the mechanical properties of the tumor microenvironment by inducing the expression and extracellular deposition of fibronectin, leading to an increased number of focal adhesions and cell spreading (49). The GP6 signaling pathway was primarily thought to be involved in platelets and their precursor megakaryocyte activation, which might shield circulating cancer cells from immunosurveillance and promote metastasis (50). It has previously been reported that the GP6 signaling pathway may play a crucial role in the metastasis of endometrial cancer and could be involved in the malignant behavior of parathyroid tumors (51, 52). In our study, GP6 signaling was enriched in the MLNM group, which supports the reliability of our results. More interestingly, 12 upregulated ECM-related proteins in this pathway were enriched. CDK5, a proline-directed serine/threonine kinase, regulates the motility and migration of tumor cells, playing a vital role in the tumor progression and metastasis in thyroid cancer (53). A growing body of evidence indicated that the VEGF signaling pathway was activated during thyroid cancer progression, particularly in LNM (54). mTOR signaling might be highly activated in aggressive histological types of thyroid cancer and represents a promising target for therapy (55). Additional evidence indicated that VEGF stimulation could activate mTOR signaling, and synchronous inhibition of the mTOR and VEGF axis impedes tumor growth and metastasis (56, 57). These findings suggested that ECM-related pathways could reflect more pronounced changes in ECM components in male than in female PTMC patients with LNM.

The clinical prognostic values of 12 upregulated DEPs between MLNM and FLNM were validated using the external TCGA-THCA and PFI data at the mRNA level. We found that multimerin 2 (MMRN2) and nidogen-2 (NID2) showed prognostic significance. Furthermore, IHC analyses were performed for validation in an independent set of PTMC patients, confirming the significant upregulation of MMRN2 and NID2 in MLNM tissues compared to FLNM tissues. MMRN2 is an endothelial-specific member of the EMI domain endowed (EDEN) protein family and the component of the ECM; it is consistently deposited along tumor capillaries and is related to neovascularization in neoplastic tissues (58, 59). Moreover, MMRN2 is upregulated in the tumor vasculature (60). Previous studies have reported that MMRN2 and CD93 colocalize in neoplastic tissues of different origins and are highly expressed during tumor angiogenesis, which is consistent with the fact that this molecule is deposited along blood vessels (60–62). Accordingly, in the present study, upregulated expression of MMRN2 in MLNM tissues correlated positively with unfavorable variables, such as metastasis and poor prognosis. NID2 is a ubiquitous component in the ECM and plays a vital role in cancer development. NID2 might regulate the progression of the tumors *via* protein digestion and absorption, focal adhesion, and ECM-receptor interactions. Previous studies demonstrated that NID2 was significantly overexpressed in many cancer tissues and positively associated with TNM stage (63, 64). The results of the present study indicated that the upregulated expression of NID2 in MLNM tumorous tissues compared to FLNM tumorous tissues correlated positively with poor survival and metastasis. Therefore, we speculate that upregulated expression of MMRN2 and NID2 in PTMC tissues might be responsible for the sex disparities in LNM. In the future, some animal experiments are needed to clarify the molecular mechanisms of MMRN2 and NID2 in the sex differences of PTMC with LNM, and a larger-sample and multicenter validation study is needed to confirm the generalizability of our results.

## Conclusions

In this study, we conducted a comprehensive proteomics comparison of thyroid tumorous tissues and produced a substantial list of DEPs that are likely related to metastasis and sex disparities. This is the first application of a direct DIA proteomics approach to explore significant sex differences in LNM in patients with PTMC. SLC25A15, DIRAS2, PLA2R1, and MTARC1 expression was downregulated in thyroid tumorous tissues with LNM compared thyroid tumorous tissues with NLNM, which was validated using the TCGA dataset. Additionally, the upregulated expression of MMRN2 and NID2 in tumorous tissues from males showed a positive relationship with unfavorable variables, such as LNMs and poor prognosis. The proteomics profiles of PTMC exhibit significant differences related to LNM and sex disparities, which furthers our understanding of the functional networks and signaling pathways related to PTMC.

## Data availability statement

The datasets presented in this study can be found in online repositories. The names of the repository/repositories and accession number(s) can be found in the article/**Supplementary Material**.

## Ethics statement

The study was approved by the Ethics Committee of PUMCH (No. JS-2670). The patients/participants provided their written informed consent to participate in this study.

## Author contributions

ZC, JW, and ZL conceived and initiated this study. ZC, ZZ, and XT performed the experiments and data analysis. RL and MW collected samples and clinical parameters. ZC prepared the figures and tables and wrote the original draft with support from JW and ZL. All authors contributed to the article and approved the submitted version.

## Funding

This research was supported by the National Nature Science Foundation of China (Nos. 32071436, 82172727, 81572459, and 31901041), Nature Science Foundation of Beijing (Nos. 7202164 and 7222127), and CAMS Innovation Fund for Medical Sciences (CIFMS) (No.2021-I2M-1-002).

## Acknowledgments

We gratefully acknowledge the helpful suggestion from our team members.

## Conflict of interest

The authors declare that the research was conducted in the absence of any commercial or financial relationships that could be construed as a potential conflict of interest.

## Publisher’s note

All claims expressed in this article are solely those of the authors and do not necessarily represent those of their affiliated organizations, or those of the publisher, the editors and the reviewers. Any product that may be evaluated in this article, or claim that may be made by its manufacturer, is not guaranteed or endorsed by the publisher.

## References

[1] Lee DH Kim YK Yu HW Choi JY Park SY Moon JH. Computed tomography for detecting cervical lymph node metastasis in patients who have papillary thyroid microcarcinoma with tumor characteristics appropriate for active surveillance. Thyroid (2019) 29(11):1653–9. doi: 10.1089/thy.2019.0100 31436140

[2] Jeon MJ Lee YM Sung TY Han M Shin YW Kim WG. Quality of life in patients with papillary thyroid microcarcinoma managed by active surveillance or lobectomy: A cross-sectional study. Thyroid (2019) 29(7):956–62. doi: 10.1089/thy.2018.0711 31038017

[3] Haugen BR Alexander EK Bible KC Doherty GM Mandel SJ Nikiforov YE. 2015 American Thyroid association management guidelines for adult patients with thyroid nodules and differentiated thyroid cancer: The American thyroid association guidelines task force on thyroid nodules and differentiated thyroid cancer. Thyroid (2016) 26(1):1–133. doi: 10.1089/thy.2015.0020 26462967 PMC4739132

[4] Kuo EJ Goffredo P Sosa JA Roman SA. Aggressive variants of papillary thyroid microcarcinoma are associated with extrathyroidal spread and lymph-node metastases: A population-level analysis. Thyroid (2013) 23(10):1305–11. doi: 10.1089/thy.2012.0563 PMC3783926 23600998

[5] Li F Wu Y Chen L Hu L Liu X. Evaluation of clinical risk factors for predicting insidious right central and posterior right recurrent laryngeal nerve lymph node metastasis in papillary thyroid microcarcinoma patients (Cn0): Experience of a single center. Ann Transl Med (2019) 7(1):8. doi: 10.21037/atm.2018.12.43 30788355 PMC6351383

[6] Zhang L Wei WJ Ji QH Zhu YX Wang ZY Wang Y. Risk factors for neck nodal metastasis in papillary thyroid microcarcinoma: A study of 1066 patients. J Clin Endocrinol Metab (2012) 97(4):1250–7. doi: 10.1210/jc.2011-1546 22319042

[7] Rahbari R Zhang L Kebebew E. Thyroid cancer gender disparity. Future Oncol (2010) 6(11):1771–9. doi: 10.2217/fon.10.127 PMC3077966 21142662

[8] Kilfoy BA Devesa SS Ward MH Zhang Y Rosenberg PS Holford TR. Gender is an age-specific effect modifier for papillary cancers of the thyroid gland. Cancer Epidemiol Biomarkers Prev (2009) 18(4):1092–100. doi: 10.1158/1055-9965.EPI-08-0976 PMC2667567 19293311

[9] Ortega J Sala C Flor B Lledo S. Efficacy and cost-effectiveness of the ultracision harmonic scalpel in thyroid surgery: An analysis of 200 cases in a randomized trial. J Laparoendosc Adv Surg Tech A (2004) 14(1):9–12. doi: 10.1089/109264204322862289 15035837

[10] Sun Y Lv H Zhang S Bai Y Shi B. Gender-specific risk of central compartment lymph node metastasis in papillary thyroid carcinoma. Int J Endocrinol (2018) 2018:6710326. doi: 10.1155/2018/6710326 29713344 PMC5866883

[11] Oh HS Park S Kim M Kwon H Song E Sung TY. Young age and Male sex are predictors of Large-volume central neck lymph node metastasis in clinical N0 papillary thyroid microcarcinomas. Thyroid (2017) 27(10):1285–90. doi: 10.1089/thy.2017.0250 28741452

[12] Kim SK Park I Woo JW Lee JH Choe JH Kim JH. Predictive factors for lymph node metastasis in papillary thyroid microcarcinoma. Ann Surg Oncol (2016) 23(9):2866–73. doi: 10.1245/s10434-016-5225-0 27075321

[13] Ucal Y Ozpinar A. Proteomics in thyroid cancer and other thyroid-related diseases: A review of the literature. Biochim Biophys Acta Proteins Proteom (2020) 1868(11):140510. doi: 10.1016/j.bbapap.2020.140510 32712303

[14] Navas-Carrillo D Rodriguez JM Montoro-Garcia S Orenes-Pinero E. High-resolution proteomics and metabolomics in thyroid cancer: Deciphering novel biomarkers. Crit Rev Clin Lab Sci (2017) 54(7-8):446–57. doi: 10.1080/10408363.2017.1394266 29084467

[15] Szklarczyk D Morris JH Cook H Kuhn M Wyder S Simonovic M. The string database in 2017: Quality-controlled protein-protein association networks, made broadly accessible. Nucleic Acids Res (2017) 45(D1):D362–D8. doi: 10.1093/nar/gkw937 PMC5210637 27924014

[16] Cancer Genome Atlas Research N. Integrated genomic characterization of papillary thyroid carcinoma. Cell (2014) 159(3):676–90. doi: 10.1016/j.cell.2014.09.050 PMC4243044 25417114

[17] Love MI Huber W Anders S. Moderated estimation of fold change and dispersion for rna-seq data with Deseq2. Genome Biol (2014) 15(12):550. doi: 10.1186/s13059-014-0550-8 25516281 PMC4302049

[18] Jiang K Li G Chen W Song L Wei T Li Z. Plasma exosomal mir-146b-5p and mir-222-3p are potential biomarkers for lymph node metastasis in papillary thyroid carcinomas. Onco Targets Ther (2020) 13:1311–9. doi: 10.2147/OTT.S231361 PMC7025673 32103998

[19] Lin P Yao Z Sun Y Li W Liu Y Liang K. Deciphering novel biomarkers of lymph node metastasis of thyroid papillary microcarcinoma using proteomic analysis of ultrasound-guided fine-needle aspiration biopsy samples. J Proteomics (2019) 204:103414. doi: 10.1016/j.jprot.2019.103414 31195151

[20] Zhan S Wang T Wang M Li J Ge W. In-depth proteomics analysis to identify biomarkers of papillary thyroid cancer patients older than 45 years with different degrees of lymph node metastases. Proteomics Clin Appl (2019) 13(5):e1900030. doi: 10.1002/prca.201900030 31148369

[21] Yi HS Chang JY Kim KS Shong M. Oncogenes, mitochondrial metabolism, and quality control in differentiated thyroid cancer. Korean J Intern Med (2017) 32(5):780–9. doi: 10.3904/kjim.2016.420 PMC5583459 28823142

[22] Chou HC Chan HL. Targeting proteomics to investigate metastasis-associated mitochondrial proteins. J Bioenerg Biomembr (2012) 44(6):629–34. doi: 10.1007/s10863-012-9466-8 22890579

[23] Chen EI. Mitochondrial dysfunction and cancer metastasis. J Bioenerg Biomembr (2012) 44(6):619–22. doi: 10.1007/s10863-012-9465-9 22892817

[24] Luo Y Ma J Lu W. The significance of mitochondrial dysfunction in cancer. Int J Mol Sci (2020) 21(16):5598. doi: 10.3390/ijms21165598 PMC7460667 32764295

[25] Jian M Yunjia Z Zhiying D Yanduo J Guocheng J. Interleukin 7 receptor activates Pi3k/Akt/Mtor signaling pathway *Via* downregulation of beclin-1 in lung cancer. Mol Carcinog (2019) 58(3):358–65. doi: 10.1002/mc.22933 30362635

[26] Li J Feng D Gao C Zhang Y Xu J Wu M. Isoforms s and l of Mrpl33 from alternative splicing have isoformspecific roles in the chemoresponse to epirubicin in gastric cancer cells *Via* the Pi3k/Akt signaling pathway. Int J Oncol (2019) 54(5):1591–600. doi: 10.3892/ijo.2019.4728 PMC6438423 30816492

[27] Bijur GN Jope RS. Rapid accumulation of akt in mitochondria following phosphatidylinositol 3-kinase activation. J Neurochem (2003) 87(6):1427–35. doi: 10.1046/j.1471-4159.2003.02113.x PMC2040497 14713298

[28] Wei F Ding L Wei Z Zhang Y Li Y Qinghua L. Ribosomal protein L34 promotes the proliferation, invasion and metastasis of pancreatic cancer cells. Oncotarget (2016) 7(51):85259–72. doi: 10.18632/oncotarget.13269 PMC5356734 27845896

[29] Ebright RY Lee S Wittner BS Niederhoffer KL Nicholson BT Bardia A. Deregulation of ribosomal protein expression and translation promotes breast cancer metastasis. Science (2020) 367(6485):1468–73. doi: 10.1126/science.aay0939 PMC7307008 32029688

[30] Lee J Seol MY Jeong S Lee CR Ku CR Kang SW. A metabolic phenotype based on mitochondrial ribosomal protein expression as a predictor of lymph node metastasis in papillary thyroid carcinoma. Med (Baltimore) (2015) 94(2):e380. doi: 10.1097/MD.0000000000000380 PMC4602546 25590838

[31] Chen F Jin Y Feng L Zhang J Tai J Shi J. Rrs1 gene expression involved in the progression of papillary thyroid carcinoma. Cancer Cell Int (2018) 18:20. doi: 10.1186/s12935-018-0519-x 29449788 PMC5812111

[32] Lee JS Adler L Karathia H Carmel N Rabinovich S Auslander N. Urea cycle dysregulation generates clinically relevant genomic and biochemical signatures. Cell (2018) 174(6):1559–70 e22. doi: 10.1016/j.cell.2018.07.019 30100185 PMC6225773

[33] Bednarz-Misa I Fleszar MG Fortuna P Lewandowski L Mierzchala-Pasierb M Diakowska D. Altered l-arginine metabolic pathways in gastric cancer: Potential therapeutic targets and biomarkers. Biomolecules (2021) 11(8):1086. doi: 10.3390/biom11081086 34439753 PMC8395015

[34] Li X Liu S Fang X He C Hu X. The mechanisms of diras family members in role of tumor suppressor. J Cell Physiol (2019) 234(5):5564–77. doi: 10.1002/jcp.27376 30317588

[35] Sutton MN Yang H Huang GY Fu C Pontikos M Wang Y. Ras-related gtpases Diras1 and Diras2 induce autophagic cancer cell death and are required for autophagy in murine ovarian cancer cells. Autophagy (2018) 14(4):637–53. doi: 10.1080/15548627.2018.1427022 PMC5959337 29368982

[36] Griveau A Devailly G Eberst L Navaratnam N Le Calve B Ferrand M. The Pla2r1-Jak2 pathway upregulates erralpha and its mitochondrial program to exert tumor-suppressive action. Oncogene (2016) 35(38):5033–42. doi: 10.1038/onc.2016.43 27041564

[37] Mitwally N Yousef E Abd Al Aziz A Taha M. Clinical significance of expression changes and promoter methylation of Pla2r1 in tissues of breast cancer patients. Int J Mol Sci (2020) 21(15):5453. doi: 10.3390/ijms21155453 PMC7432085 32751713

[38] Brennan K Holsinger C Dosiou C Sunwoo JB Akatsu H Haile R. Development of prognostic signatures for intermediate-risk papillary thyroid cancer. BMC Cancer (2016) 16(1):736. doi: 10.1186/s12885-016-2771-6 27633254 PMC5025616

[39] Plitzko B Ott G Reichmann D Henderson CJ Wolf CR Mendel R. The involvement of mitochondrial amidoxime reducing components 1 and 2 and mitochondrial cytochrome B5 in n-reductive metabolism in human cells. J Biol Chem (2013) 288(28):20228–37. doi: 10.1074/jbc.M113.474916 PMC3711290 23703616

[40] Rixen S Havemeyer A Tyl-Bielicka A Pysniak K Gajewska M Kulecka M. Mitochondrial amidoxime-reducing component 2 (Marc2) has a significant role in n-reductive activity and energy metabolism. J Biol Chem (2019) 294(46):17593–602. doi: 10.1074/jbc.RA119.007606 PMC6873197 31554661

[41] Wu D Wang Y Yang G Zhang S Liu Y Zhou S. A novel mitochondrial amidoxime reducing component 2 is a favorable indicator of cancer and suppresses the progression of hepatocellular carcinoma by regulating the expression of P27. Oncogene (2020) 39(38):6099–112. doi: 10.1038/s41388-020-01417-6 PMC7498369 32811980

[42] Winkler J Abisoye-Ogunniyan A Metcalf KJ Werb Z. Concepts of extracellular matrix remodelling in tumour progression and metastasis. Nat Commun (2020) 11(1):5120. doi: 10.1038/s41467-020-18794-x 33037194 PMC7547708

[43] Najafi M Farhood B Mortezaee K. Extracellular matrix (Ecm) stiffness and degradation as cancer drivers. J Cell Biochem (2019) 120(3):2782–90. doi: 10.1002/jcb.27681 30321449

[44] Ran T Chen Z Zhao L Ran W Fan J Hong S. Lamb1 is related to the T stage and indicates poor prognosis in gastric cancer. Technol Cancer Res Treat (2021) 20:15330338211004944. doi: 10.1177/15330338211004944 33784890 PMC8020091

[45] Liang J Li H Han J Jiang J Wang J Li Y. Mex3a interacts with Lama2 to promote lung adenocarcinoma metastasis *Via* Pi3k/Akt pathway. Cell Death Dis (2020) 11(8):614. doi: 10.1038/s41419-020-02858-3 32792503 PMC7427100

[46] Kunitomi H Kobayashi Y Wu RC Takeda T Tominaga E Banno K. Lamc1 is a prognostic factor and a potential therapeutic target in endometrial cancer. J Gynecol Oncol (2020) 31(2):e11. doi: 10.3802/jgo.2020.31.e11 31912669 PMC7044014

[47] Galatenko VV Maltseva DV Galatenko AV Rodin S Tonevitsky AG. Cumulative prognostic power of laminin genes in colorectal cancer. BMC Med Genomics (2018) 11(Suppl 1):9. doi: 10.1186/s12920-018-0332-3 29504916 PMC5836818

[48] He L Yuan L Sun Y Wang P Zhang H Feng X. Glucocorticoid receptor signaling activates Tead4 to promote breast cancer progression. Cancer Res (2019) 79(17):4399–411. doi: 10.1158/0008-5472.CAN-19-0012 31289134

[49] Sorrentino G Ruggeri N Zannini A Ingallina E Bertolio R Marotta C. Glucocorticoid receptor signalling activates yap in breast cancer. Nat Commun (2017) 8:14073. doi: 10.1038/ncomms14073 28102225 PMC5253666

[50] Kopp HG Placke T Salih HR. Platelet-derived transforming growth factor-beta down-regulates Nkg2d thereby inhibiting natural killer cell antitumor reactivity. Cancer Res (2009) 69(19):7775–83. doi: 10.1158/0008-5472.CAN-09-2123 19738039

[51] Yadav VK Lee TY Hsu JB Huang HD Yang WV Chang TH. Computational analysis for identification of the extracellular matrix molecules involved in endometrial cancer progression. PloS One (2020) 15(4):e0231594. doi: 10.1371/journal.pone.0231594 32315343 PMC7173926

[52] Condello V Cetani F Denaro M Torregrossa L Pardi E Piaggi P. Gene expression profile in metastatic and non-metastatic parathyroid carcinoma. Endocr Relat Cancer (2021) 28(2):111–34. doi: 10.1530/ERC-20-0450 33290252

[53] Pozo K Castro-Rivera E Tan C Plattner F Schwach G Siegl V. The role of Cdk5 in neuroendocrine thyroid cancer. Cancer Cell (2013) 24(4):499–511. doi: 10.1016/j.ccr.2013.08.027 24135281 PMC3849320

[54] Du Y Zhu J Chu BF Yang YP Zhang SL. Mir-548c-3p suppressed the progression of papillary thyroid carcinoma *Via* inhibition of the Hif1alpha-mediated vegf signaling pathway. Eur Rev Med Pharmacol Sci (2019) 23(15):6570–8. doi: 10.26355/eurrev_201908_18543 31378898

[55] Kouvaraki MA Liakou C Paraschi A Dimas K Patsouris E Tseleni-Balafouta S. Activation of mtor signaling in medullary and aggressive papillary thyroid carcinomas. Surgery (2011) 150(6):1258–65. doi: 10.1016/j.surg.2011.09.022 22136849

[56] Yan B Kong M Chen S Chen YH. Vegf stimulation enhances livin protein synthesis through mtor signaling. J Cell Biochem (2010) 111(5):1114–24. doi: 10.1002/jcb.22797 20717925

[57] Pal K Madamsetty VS Dutta SK Wang E Angom RS Mukhopadhyay D. Synchronous inhibition of mtor and Vegf/Nrp1 axis impedes tumor growth and metastasis in renal cancer. NPJ Precis Oncol (2019) 3:31. doi: 10.1038/s41698-019-0105-2 31840081 PMC6895165

[58] Andreuzzi E Colladel R Pellicani R Tarticchio G Cannizzaro R Spessotto P. The angiostatic molecule multimerin 2 is processed by mmp-9 to allow sprouting angiogenesis. Matrix Biol (2017) 64:40–53. doi: 10.1016/j.matbio.2017.04.002 28435016

[59] Christian S Ahorn H Novatchkova M Garin-Chesa P Park JE Weber G. Molecular cloning and characterization of endoglyx-1, an emilin-like multisubunit glycoprotein of vascular endothelium. J Biol Chem (2001) 276(51):48588–95. doi: 10.1074/jbc.M106152200 11559704

[60] Noy PJ Lodhia P Khan K Zhuang X Ward DG Verissimo AR. Blocking Clec14a-Mmrn2 binding inhibits sprouting angiogenesis and tumour growth. Oncogene (2015) 34(47):5821–31. doi: 10.1038/onc.2015.34 PMC4724939 25745997

[61] Galvagni F Nardi F Spiga O Trezza A Tarticchio G Pellicani R. Dissecting the Cd93-multimerin 2 interaction involved in cell adhesion and migration of the activated endothelium. Matrix Biol (2017) 64:112–27. doi: 10.1016/j.matbio.2017.08.003 28912033

[62] Randles MJ Humphries MJ Lennon R. Proteomic definitions of basement membrane composition in health and disease. Matrix Biol (2017) 57-58:12–28. doi: 10.1016/j.matbio.2016.08.006 27553508

[63] Yu ZH Wang YM Jiang YZ Ma SJ Zhong Q Wan YY. Nid2 can serve as a potential prognosis prediction biomarker and promotes the invasion and migration of gastric cancer. Pathol Res Pract (2019) 215(10):152553. doi: 10.1016/j.prp.2019.152553 31362888

[64] Shan Z Wang W Tong Y Zhang J. Genome-scale analysis identified Nid2, sparc, and Mfap2 as prognosis markers of overall survival in gastric cancer. Med Sci Monit (2021) 27:e929558. doi: 10.12659/MSM.929558 33758160 PMC8006563

