# Supplementary material for: Comprehensive analysis of tissue proteomics in patients with papillary thyroid microcarcinoma uncovers the underlying mechanism of lymph node metastasis and its significant sex disparities
Source: Front Oncol. 2022 Aug 29;12:887977. doi: 10.3389/fonc.2022.887977 (PMC9465038; doi:10.3389/fonc.2022.887977)

Supplementary Figure 1: Gene Ontology (GO) enrichment and pathway enrichment analysis of DEPs between the LNM and NLNM groups. (A-C) GO analysis revealed enrichment of downregulated DEPs in the biological process (BP), cellular component (CC) and molecular function (MF) categories. (D-F) GO analysis revealed enrichment of upregulated DEPs in the BP, CC, and MF categories. (G) pathway enrichment analysis of downregulated DEPs between LNM and NLNM. (H) pathway enrichment analysis of upregulated DEPs between LNM and NLNM

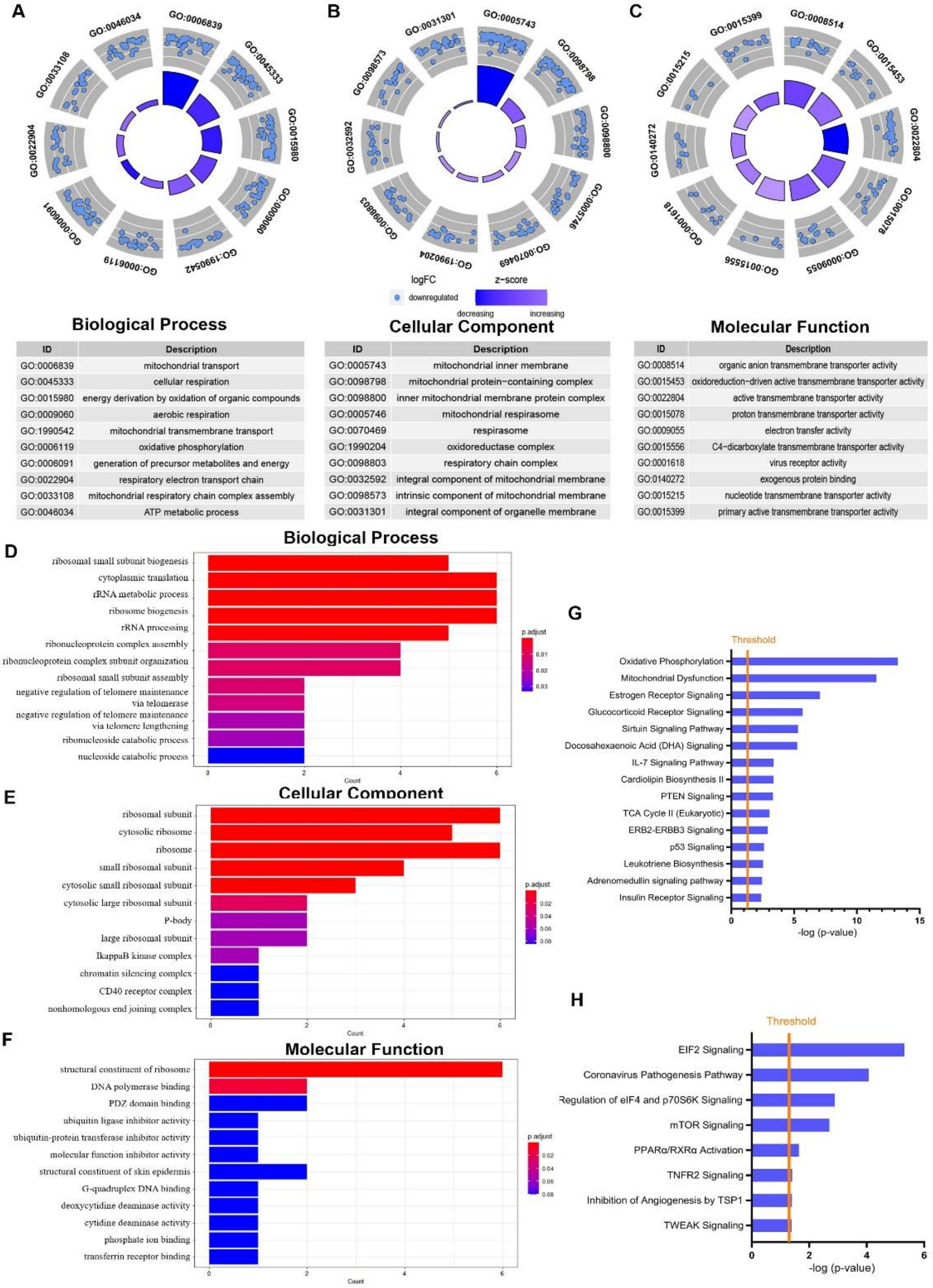

Supplementary Figure 2: Differential expression of SLC25A15 (A) and PLA2R1 (B) between neoplastic and peritumoral tissues. IHC staining was assessed as the sum of the percentage and intensity scores, showing SLC25A15 and PLA2R1 to be significantly upregulated in neoplastic versus peritumoral tissues (*P < 0.05, ***P < 0.001). Representative immunohistochemical staining of SLC25A15 (C, neoplastic; D, peritumoral) and PLA2R1 (E, neoplastic; F, peritumoral) in thyroid tissues.

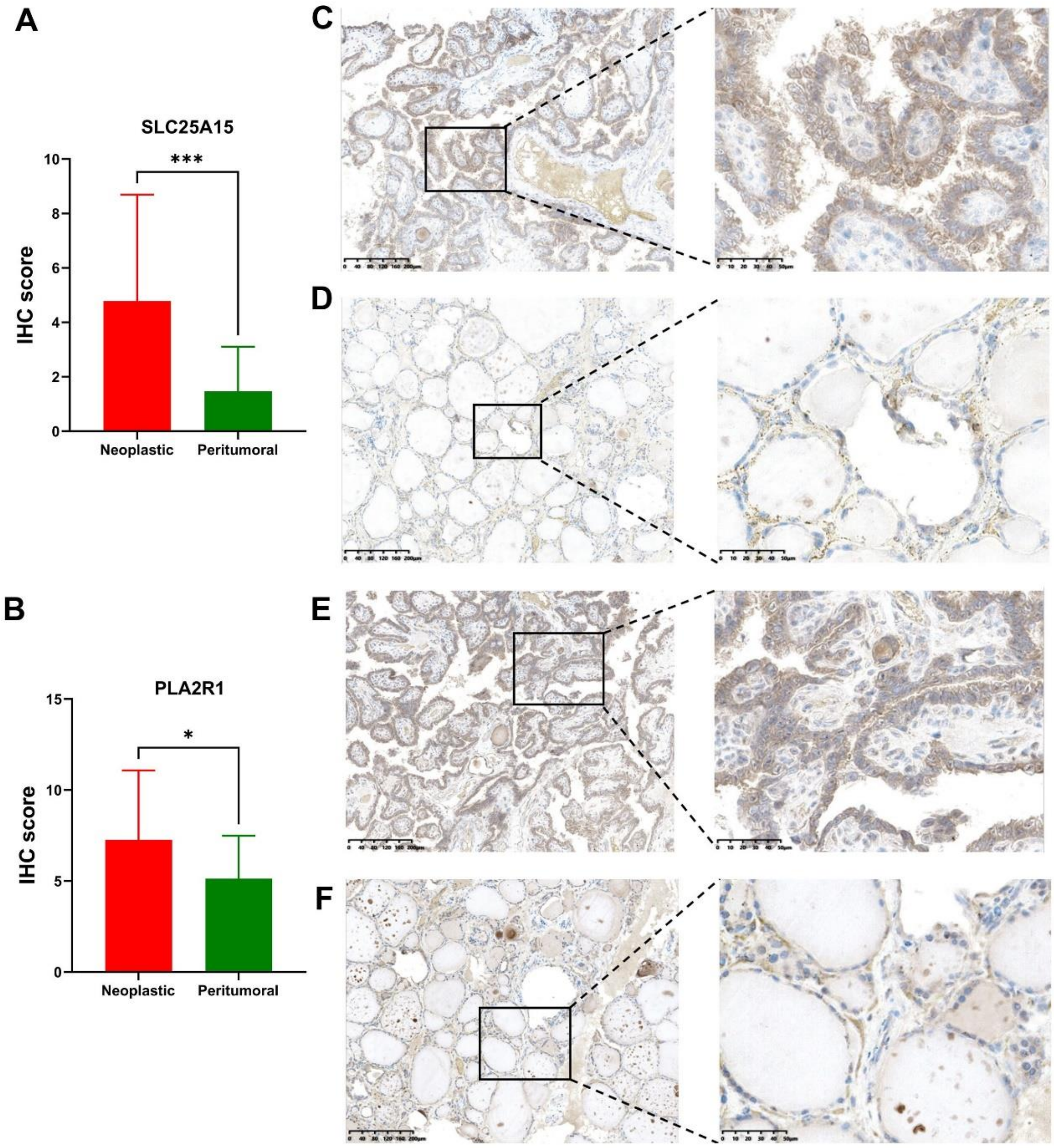

Supplementary Figure 3: Gene Ontology (GO) enrichment and pathway enrichment analysis of DEPs between the MLNM and FLNM groups. (A-C) GO analysis revealed enrichment of upregulated DEPs in the biological process (BP), cellular component (CC) and molecular function (MF) categories. (D-F) GO analysis revealed enrichment of downregulated DEPs in the BP, CC, and MF categories. (G) pathway enrichment analysis of downregulated DEPs between MLNM and FLNM. (H) pathway enrichment analysis of upregulated DEPs between MLNM and FLNM

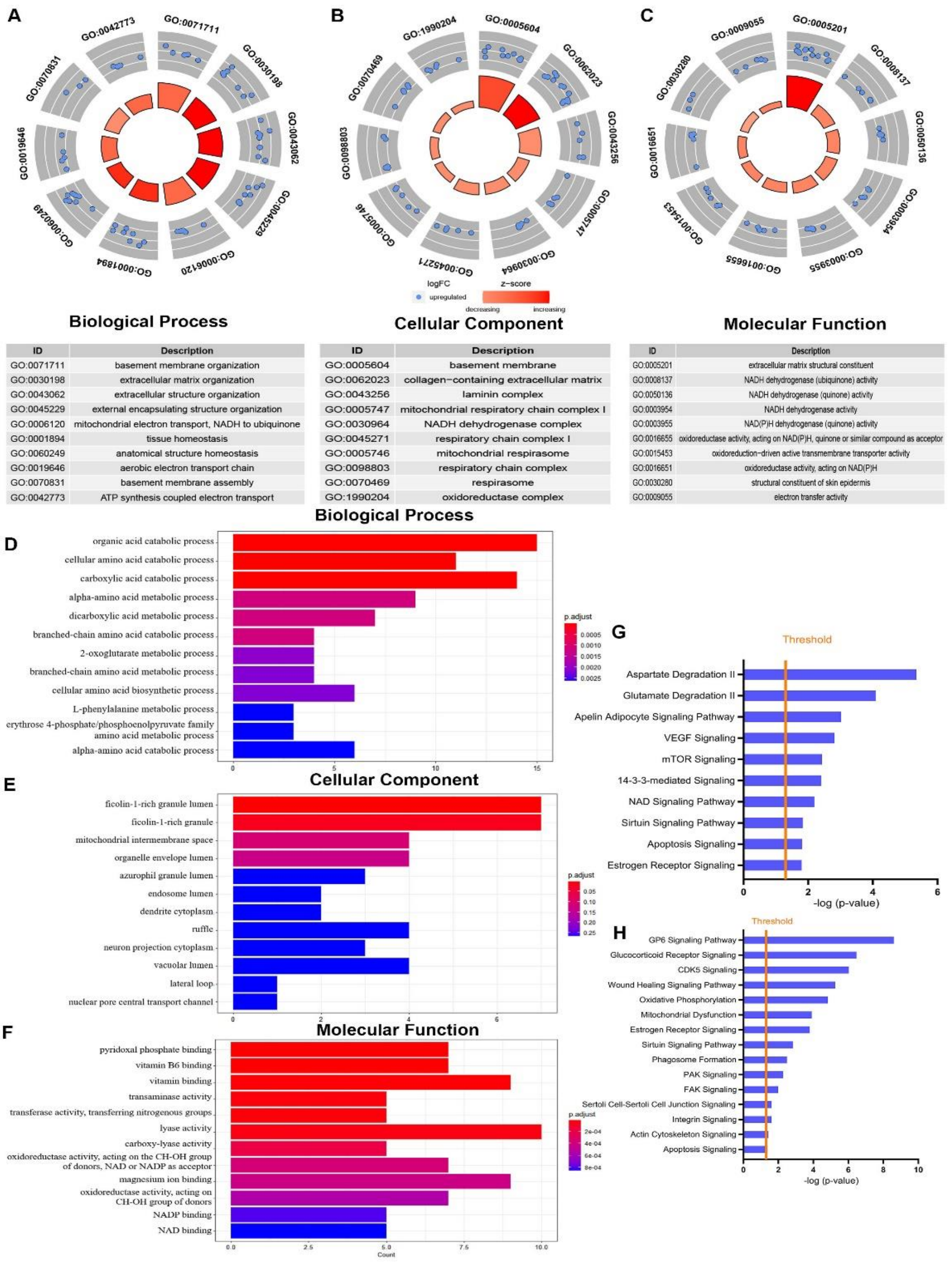

Supplement: Supplementary file 7 [file DataSheet_1.pdf]
